# The Comparative Survey of Coordinated Regulation of Steroidogenic Pathway in Japanese Flounder (*Paralichthys olivaceus*) and Chinese Tongue Sole (*Cynoglossus semilaevis*)

**DOI:** 10.3390/ijms23105520

**Published:** 2022-05-15

**Authors:** Fan Yang, Yapeng Wang, Wei Lu, Wenyu Zong, Qing Zhu, Jie Cheng

**Affiliations:** 1 Key Laboratory of Marine Genetics and Breeding (Ocean University of China), Ministry of Education, 5 Yushan Road, Qingdao 266003, China; yangfan4303@stu.ouc.edu.cn (F.Y.); wangyapeng@stu.ouc.edu.cn (Y.W.); lw1981@ouc.edu.cn (W.L.); zongwenyu@stu.ouc.edu.cn (W.Z.); zhuqing@stu.ouc.edu.cn (Q.Z.); 2 Key Laboratory of Tropical Aquatic Germplasm of Hainan Province, Sanya Oceanographic Institution, Ocean University of China, Sanya 572024, China; 3 Laboratory for Marine Fisheries Science and Food Production Processes, Pilot National Laboratory for Marine Science and Technology (Qingdao), 1 Wenhai Road, Qingdao 266237, China

**Keywords:** steroidogenesis, hypothalamic-pituitary-gonadal axis, regulatory network, *Paralichthys olivaceus*, *Cynoglossus semilaevis*

## Abstract

Steroidogenesis controls the conversion of cholesterol into steroid hormones through the complex cascade reaction of various enzymes, which play essential roles in sexual differentiation and gonadal development in vertebrates, including teleosts. Japanese flounder (*Paralichthys olivaceus*) and Chinese tongue sole (*Cynoglossus semilaevis*) are important marine cultured fishes in China and have remarkable sexual dimorphism with bigger females and sex reversal scenarios from female to neo-male. Several steroidogenic genes have been analyzed individually in the two species, but there is a lack of information on the coordinated interaction of steroidogenic gene regulation. Therefore, in this study, through genomic and transcriptomic analysis, 39 and 42 steroidogenic genes were systematically characterized in *P. olivaceus* and *C. semilaevis* genomes, respectively. Phylogenetic and synteny analysis suggested a teleost specific genome duplication origin for *cyp19a1a*/*cyp19a1b*, *hsd17b12a*/*hsd17b12b*, *ara*/*arb* and *esr2a*/*esr2b* but not for *star*/*star2* and *cyp17a1*/*cyp17a2*. Comparative transcriptome analysis revealed conserved expression patterns for steroidogenic genes in *P. olivaceus* and *C. smilaevis* gonads; *star*/*star2*, *cyp11a*/*cyp11c*, *cyp17a1*/*cyp17a2*, *cyp21a*, *hsd3b1*, *hsd11b* and *hsd20b* were strongly expressed in testis, while *cyp19a1a* and *hsd17b* genes were highly expressed in ovaries. Only a few genes were differentially expressed between male and neo-male testis of both *P. olivaceus* and *C. semilaevis*, and even fewer genes were differentially regulated in the brains of both species. Network analysis indicated that *cyp11c*, *cyp17a1* and *hsd3b1* actively interacted with other steroidogenic genes in *P. olivaceus* and *C. semilaevis*, and may play a more sophisticated role in the steroid hormone biosynthesis cascade. The coordinated interaction of steroidogenic genes provided comprehensive insights into steroidogenic pathway regulation with a global biological impact, as well as sexual development in teleost species.

## 1. Introduction

Steroidogenesis is the conversion of cholesterol into biologically active steroid hormones by the complex cascade reaction of various enzymes, which plays essential roles during many physiological processes including sexual differentiation, gonadal development, growth and maturation [1]. Steroidogenesis occurs primarily in tissues, such as the gonads, interrenal gland and brain, which is, in general, controlled by the hypothalamus–pituitary–interrenal (HPI) and hypothalamus–pituitary–gonadal (HPG) axes and has a considerable impact on regulating complex steroidogenic process in vertebrates, and further affects reproduction [2,3].

In vertebrates, including teleosts, steroidogenesis is triggered by the mobilization of cholesterol by steroidogenic acute regulatory protein (star) from the outer to the inner mitochondrial membrane [4,5]. The following sophisticated process recruits diverse enzymes, such as cytochrome P450 and hydroxysteroid dehydrogenase, for the synthesis of C18 estradiol, C19 testosterone and C21 cortisol [6]. The cytochrome P450 (cyp) is a multigene family that is involved in catalyzing the production of sexual steroids [7,8]. For example, cyp11a is the enzyme that catalyzes the generation of pregnenolone, which is the primary step in the initiation of steroid hormone biosynthesis [6,9]. Cyp11b/c can promote the production of corticosterone, cortisol and 11β-OH-testosterone [10,11], which has a great impact on male ontogenesis. Cyp17a1 and cyp17a2 are two isoforms of steroid 17α-hydroxylase/17,20-lyase [12], with cyp17a1 possessing both 17α-hydroxylase and 17,20-lyase activities, while cyp17a2 has 17α-hydroxylase activity. Moreover, cyp17a1 was proven to participate in generating ovary estradiol, while the generation of C21 steroids, such as cortisol, in the head kidney is associated with cyp17a2 [13,14]. Aromatase (cyp19a1) is responsible for the transformation of androstenedione into estrone, and testosterone into estradiol, in which the up- and down-regulation of *cyp19a1* are crucial for ovarian and testicular differentiation [15]. In addition, Hydroxysteroid dehydrogenases (hsds) are also involved in the regulation of steroid hormone biosynthesis [16]. 3-beta-hydroxysteroid dehydrogenase (hsd3b) is important in the production of progesterone and testosterone [17]. Corticosteroid 11-beta-dehydrogenase isozyme 2 (hsd11b2) was found to exert a major influence on the testicular differentiation of catfish (*Clarias gariepinus*) [18]. In zebrafish (*Danio rerio*), hsd20b2 is able to synthesize 20β-dihydrocortisone from cortisone [19], and *hsd17b3* is highly expressed in the ovary [20]. In addition, sex hormone receptors, such as estrogen (esr) and androgen (ar) receptors, are also important for the regulation of sex steroid hormones [6,21,22], which regulate reproductive activities by combining with esr and ar [21].

Teleost species exhibit a wide range of reproductive strategies for sex determination and differentiation, and steroids are required during all stages of the reproductive cycle in teleosts which include sex differentiation, maturation, growth and sexual behavior [5]. Japanese flounder (*Paralichthys olivaceus*) and Chinese tongue sole (*Cynoglossus semilaevis*), both belonging to Pleuronectiformes, are important maricultured fish species in China, Japan and Korea [23,24]. *P. olivaceus* and *C. semilaevis* have remarkable sexual dimorphism, with females growing faster and bigger than males; they also present a sex reversal scenario from female to neo-male during the key period of sex differentiation [25,26]. Therefore, it makes the sex control for all female production more economic and beneficial in aquaculture [25]. The steroidogenic pathway, important for sex hormone homeostasis, has been proven to play essential roles in gonadal development and sex differentiation in *P. olivaceus* and *C. semilaevis*, especially for the sex reversal of both species [26,27,28]. Many steroidogenic genes, such as *star* [29], *cyp11* [9,10], *cyp17a* [30], *cyp19a1* [28], as well as the *hsd* family [24] and sex hormone receptors [31], have been characterized in *P. olivaceus* and *C. semilaevis*. However, most of these studies are only focused on a limited number of steroidogenic genes rather than the full complement of the steroidogenic pathways that comprise the coordinated cascade reaction in steroid biosynthesis. Therefore, in this study, the steroidogenic genes in *P. olivaceus* and *C. semilaevis* were characterized, and their evolution, expression and coordinated interaction were compared through molecular evolution and transcriptome analysis, which will provide a comprehensive understanding of the relationships between the regulation of steroidogenic pathways and sexual development in teleost species.

## 2. Results and Discussion

### 2.1. Genomic Landscape, Functional Domain and Phylogeny of Steroidogenic Genes in P. olivaceus and C. semilaevis

**Figure 1:**
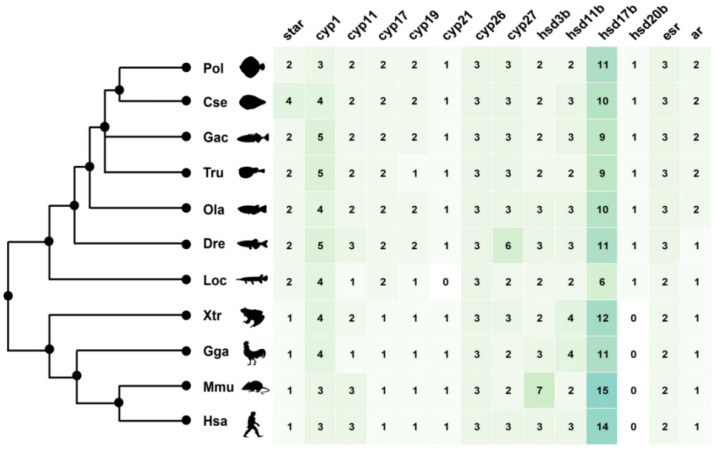
Copy number of steroidogenic genes in selected vertebrate genomes presented as a heatmap along with their phylogeny. The color background represents the gene number. Abbreviations as follows: Pol: *Paralichthys olivaceus*; Cse: *Cynoglossus semilaevis*; Gac: *Gasterosteus aculeatus*; Tru: *Takifugu rubripes*; Ola: *Oryzias latipes*; Dre: *Danio rerio*; Loc: *Lepisosteus oculatus*; Xtr: *Xenopus tropicalis*; Gga: *Gallus gallus*; Mmu: *Mus musculus*; Hsa: *Homo sapiens*.

Through a genome-wide screen, 39 and 42 genes participating in steroid biosynthesis were identified in the *P. olivaceus* and *C. semilaevis* genomes, respectively (Figure 1 and Table S1), including *star*, *cyp*, *hsd* genes, as well as steroid receptors genes *esr* and *ar* (Figure 1 and Table S2). The copy number of these steroidogenic genes between tetrapod and teleost lineages was generally conserved with several gene duplication events observed in the teleost lineage (Table S1), most likely originating from the teleost specific genome duplication (TSGD).

**Figure 2:**
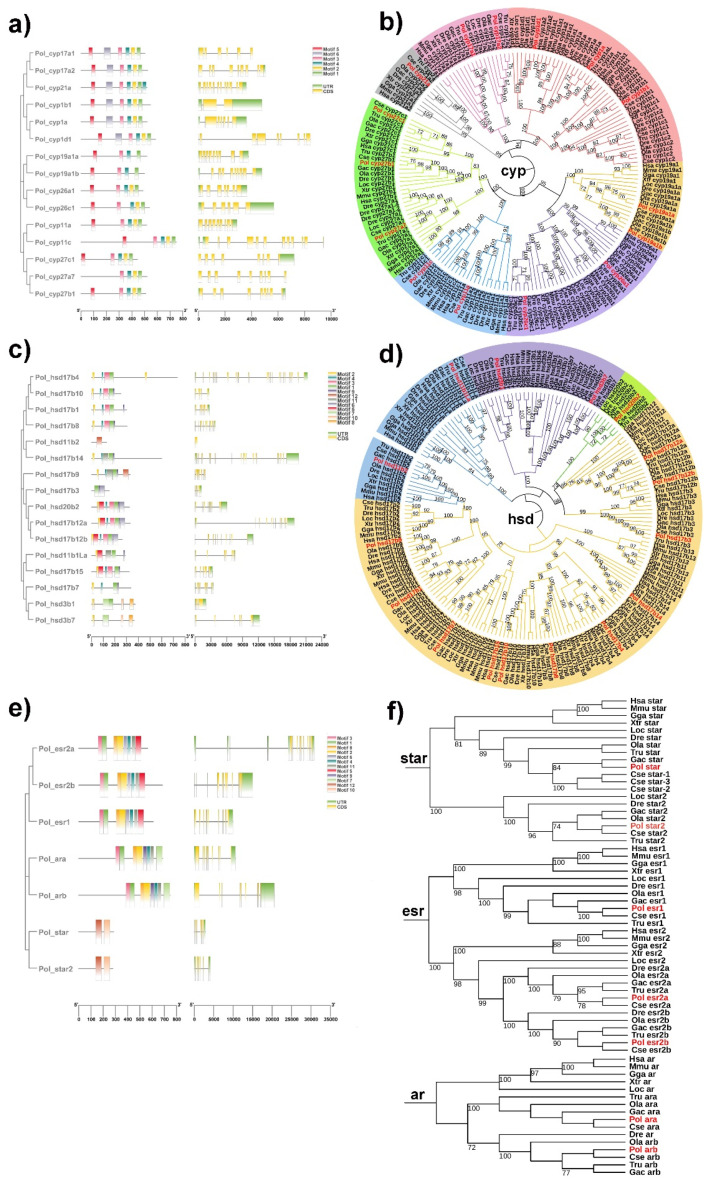
Characterization of steroidogenic proteins. The conserved motif and structure of (**a**) cyp, (**c**) hsd, (**e**) star, esr and ar in *P. olivaceus*, and the phylogeny of (**b**) cyp, (**d**) hsd, (**f**) star, esr and ar in selected vertebrates. The phylogenic trees were constructed with a bootstrap of 1000 times. The sub-families of cyp and hsd were labeled with different background colors, and the *P. olivaceus* proteins were highlighted in red.

Specifically, 16 and 17 *cyp* genes were obtained in the *P. olivaceus* and *C. semilaevis* genomes, respectively. There were comparable numbers of *cyp1*, *cyp11*, *cyp21*, *cyp26* and *cyp27* genes between tetrapods and teleosts, whereas two *cyp17*s and two *cyp19*s were identified in teleosts but only one copy was found in tetrapods, indicating their putative TSGD origin in teleost lineages (Figure 1 and Table S1). Five conserved motifs (motif 1–5) were shared among *P. olivaceus cyp* genes with motif 6 only present in *cyp1*s, *cyp17*s and *cyp21*, which was consistent with their phylogenetic relationships (Figure 2a,b). Moreover, the vertebrate *cyp* genes were clustered into seven clades, corresponding to the *cyp1*, *cyp11*, *cyp17*, *cyp19*, *cyp21*, *cyp26* and *cyp27* families (Figure 2b). The *cyp17* clade was further divided into two branches with the teleost *cyp17a1*s clustered with orthologous of tetrapods, while teleost *cyp17a2*s were further clustered together (Figure 2b). The *cyp19a1* clade was also grouped into two branches, which corresponded to *cyp19a1* of tetrapods and *cyp19a1a*/*cyp19a1b* of teleosts, indicating their TSGD origin in teleost lineages.

*Hsd3b*, *hsd11b*, *hsd17b* and *hsd20b* were pivotal members of the *hsd* genes. In total, 16 *hsd* genes were obtained in the *P. olivaceus* and *C. semilaevis* genomes, respectively, which were generally conserved between tetrapods and teleosts, with *hsd20b* only present in teleosts but not in tetrapods (Figure 1). Two or three *hsd3b* genes were obtained in most selected vertebrates as the conserved *hsd3b1* and *hsd3b7*, while *hsd3b1*, *-2*, *-3*, *-4*, *-5*, *-6* only existed in mice (Table S2). *Hsd17b* was the largest *hsd* family, with 11 and 10 *hsd17b* genes from *P. olivaceus* and *C. semilaevis*, respectively (Figure 1). The functional domain of *P. olivaceus hsd* genes was less conserved with 12 motifs detected (Figure 2c). Moreover, *hsd17b12a* and *hsd17b12b* were clustered into two branches in teleosts and further clustered with tetrapod *hsd17b12s*, suggesting a genome duplication origin in teleosts from the ancestor *hsd17b12* (Figure 2d).

In addition, a single copy of *star* was present in tetrapods while two copies were identified in most teleost species as *star* and *star2* (Figure 1). Interestingly, three copies of *star* existed in *C. semilaevis* (Table S2), with *star-2* and *star-3* located on Z and W chromosomes of *C. semilaevis*, respectively. With regard to *esr*, *esr1* was conserved as a single copy among selected vertebrates, whereas there were two *esr2s*, *esr2a* and *esr2b*, in most teleost species (Table S2). This was also found in *ar* genes, with *ara* and *arb* present in the teleost lineages (Table S2). In *P. olivaceus*, the functional motifs of *esr*, *ar* and *star* genes were generally conserved. As sex hormone receptors, *esr* and *ar* shared seven motifs, with only motif 5 and motif 7 being specific to their C-terminals, respectively (Figure 2e). Moreover, *star* and *star2* were split into two clades, which was similar to the findings of Yu et al. [32]. *Esr*s were also clustered into two clades, as *esr1* and *esr2*, with *esr2* further divided into two branches (Figure 2f), as *esr2* in tetrapods and *esr2a* and *esr2b* in teleosts.

### 2.2. The Duplication of Steroidogenic Genes in the Teleost Lineages

Teleost fishes and humans show remarkable conservation in many developmental and physiological aspects, including the endocrine system and steroid hormone related processes [6]. Here, to address the impact of genome duplication in teleosts on steroid hormone biosynthesis, seven pairs of steroidogenic genes, including *star*/*star2*, *cyp11a*/*cyp11c*, *cyp17a1*/*cyp17a2*, *cyp19a1a*/*cyp19a1b*, *hsd17b12a*/*hsd17b12b*, *esr2a*/*esr2b* and *ara*/*arb*, were employed for the synteny analysis.

**Figure 3:**
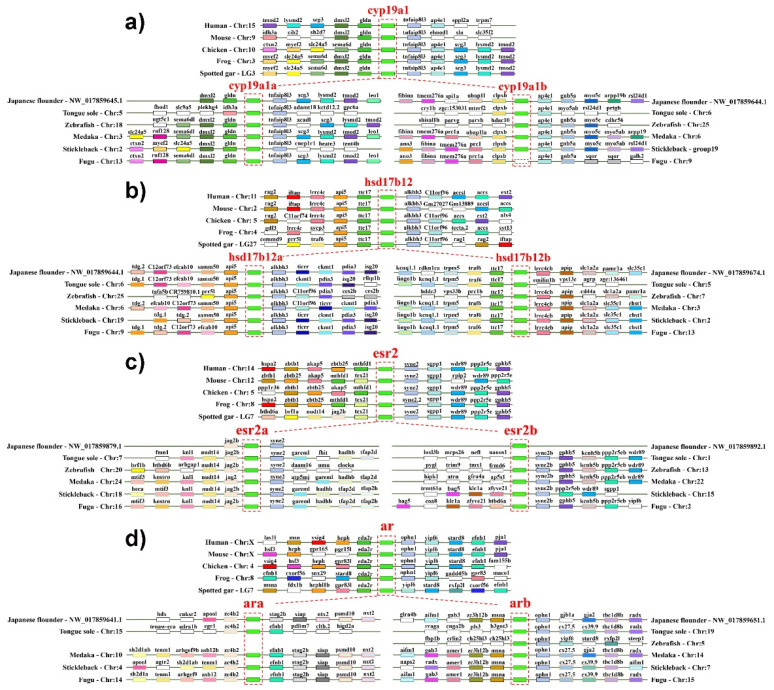
Synteny analysis of putatively duplicated steroidogenic genes between teleost and tetrapod lineages according to the TSGD, as (**a**) cyp19a1, (**b**) hsd17b12, (**c**) esr2 and (**d**) ar. Genes were shown as colored boxes with their names on top. The boxes with the same color were orthologous among different species.

Firstly, there was conserved synteny between *cyp19a1a* and *cyp19a1b* in vertebrates. For example, conservative upstream (*gldn*, *dmxl2*) and downstream (*tnfaip8l3*) genes were found in both *cyp19a1* and *cyp19a1a*, while *ap4e1* was adjacent to *cyp19a1* and *cyp19a1b* (Figure 3a). It was noteworthy that *cyp19a1b* was absent in fugu, but the conserved neighboring genes and chromosomes can still be identified (Figure 3a). This indicated that *cyp19a1a* and *cyp19a1b* originated from TSGD, which supported the previous conclusion [33]. In addition, both *hsd17b12a* and *hsd17b12b* in teleosts were found to share conserved adjacent genes with *hsd17b12*, such as *alkbh3* and *api5* between *hsd17b12* of higher vertebrates and *hsd17b12a* of teleosts, and *ttc17* and *lrrc4c* between *hsd17b12* and *hsd17b12b* (Figure 3b). Consistent with the phylogeny, *esr2* in tetrapods shared the common gene *syne2* with teleost *esr2a* and *esr2b* (Figure 3c). Moreover, *esr2* and *esr2b* were more conservative with neighboring genes *wdr89*, *ppp2r5e* and *gphb5* (Figure 3c). Similarly, *ar* and *ara* contained the conserved neighboring gene *efnb1*, while *ophn1* was the common gene between *ar* and *arb* (Figure 3d), consistent with their phylogeny (Figure 2f). Together with the results of phylogeny and synteny, it suggested that *cyp19a1a*/*1b*, *hsd17b12a*/*b*, *ara*/*b* and *esr2a*/*2b* in teleosts indeed originated from the TSGD.

On the other hand, the synteny of *star* between tetrapods and teleosts was generally conserved but not with *star2* of teleosts (Figure S1a). For example, *grk5*, *lsm1* and *bag4* were conserved downstream genes of *star*, with *grk5* absent in humans, mice and chickens. Upstream genes of *star* were less conserved, with *ash2l* existing only in tetrapods and spotted gar (Figure S1a). This indicated that although two *star* genes were present in teleosts, *star2* probably originated independently in the teleost lineage during evolution. This also can be seen in *cyp11*s and *cyp17a*s (Figure S1b,c). For instance, no conserved upstream and downstream genes were identified between *cyp17a1* and *cyp17a2* in teleosts, proving that they did not originate from the TSGD (Figure S1c), although *cyp17a1* had conserved downstream genes between tetrapods and teleosts, such as *borcs7* and *nt5c2*. Moreover, the phylogeny of *cyp17a1* between tetrapods and teleosts was closer than that of *cyp17a1* and *cyp17a2* (Figure 2b), indicating that *cyp17a1* and *cyp17a2* in teleost species were probably gained independently during evolution. Therefore, it revealed that *star*/*star2*, *cyp11a*/*11c* and *cyp17a1*/*17a2* did not originate from TSGD as previously assumed [6].

### 2.3. Evolutionary Dynamics of Steroidogenic Genes in Teleost Lineages

**Figure 4:**
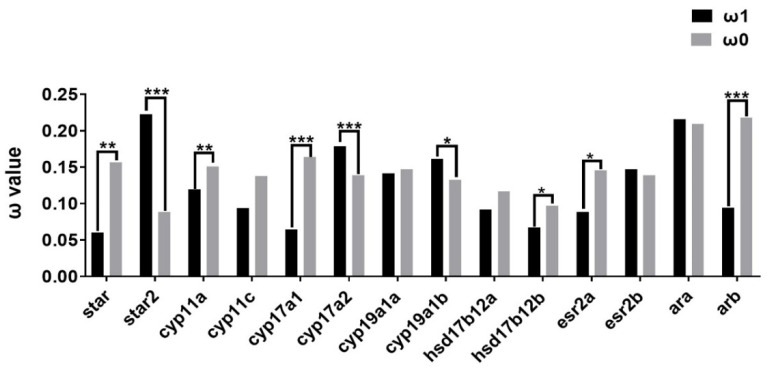
Molecular evolution of the putatively duplication originated steroidogenic genes in teleost lineages with branch model tests. Black and grey columns indicate ω values of the foreground branch and background branch, respectively. * indicates *p* < 0.05, ** indicate *p* < 0.01, *** indicate *p* < 0.001.

To further understand the evolutionary dynamics of steroidogenic pathways in teleost lineages, the ratio of non-synonymous substitution to synonymous substitution (ω, dN/dS) was analyzed to detect the possibility of selection. Site model (SM) tests showed generally conserved ω values (0–0.4) among teleost steroidogenic genes (Table S3 and Figure S2a), indicating their functional conservation during evolution. Specifically, *hsd20b2* represented the highest ω values (0.3941), while *cyp26b1* showed the lowest (0.0291) (Figure S2a). There were also diverse ω values in closely related genes, such as between *star* and *star2*, *cyp26a1*/*b1*/*c1*, *hsd11b1L* and *hsd11b2*, and *hsd17b* genes (Figure S2a). In branch model (BM) tests, the ω values of nine genes were significantly different between *P. olivaceus* and other teleost species (Figure S2b), among which seven genes of *cyp11c*, *cyp21*, *cyp27c1*, *hsd11b1L*, *hsd17b4*, *hsd17b8* and *hsd17b14* exhibited higher ω values in *P. olivaceus* than in other teleost species, indicating greater selection pressure in *P. olivaceus* (Figure S2b). Moreover, among the putatively duplicated steroidogenic genes, BM tests indicated varied evolutionary patterns. For instance, in teleost lineages, *star2*, *cyp17a2* and *cyp19a1b* showed a faster evolutionary rate (ω1) than their paralogues (ω0) (Figure 4), while other TSGD originated genes (*hsd17b12a*/*b*, *ara*/*b* and *esr2a*/*2b*) do not represent elevated evolution (Figure 4), indicating possible divergence independent from the TSGD.

### 2.4. Expression Profiles of Steroidogenic Genes in P. olivaceus and C. semilaevis

**Figure 5:**
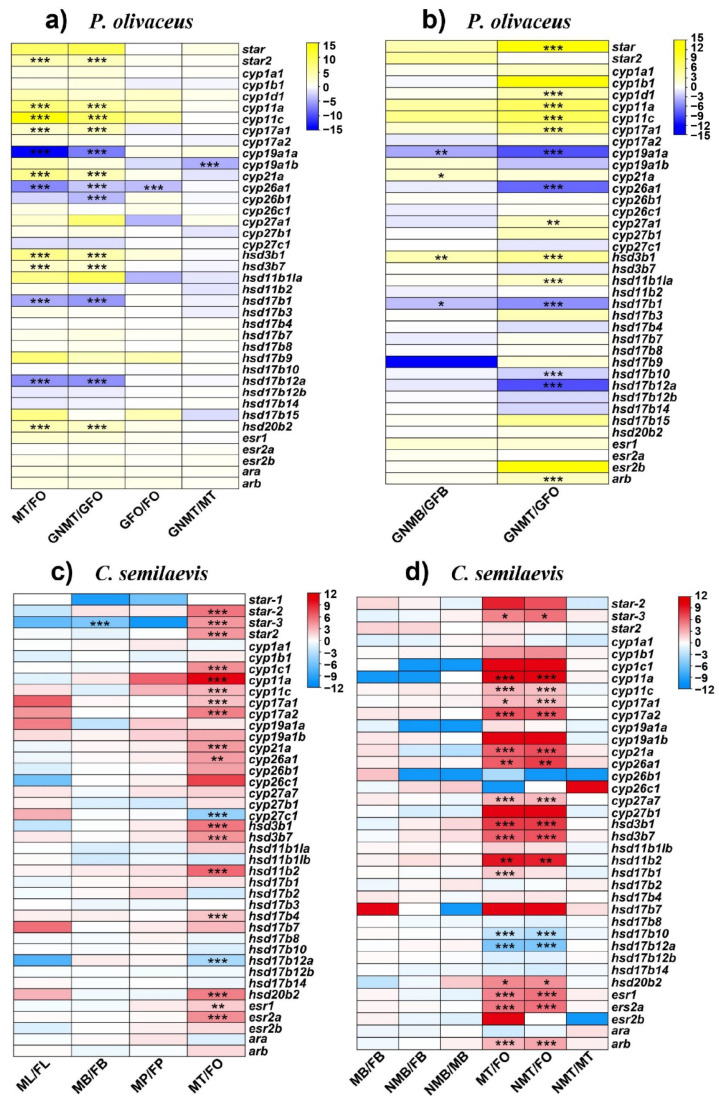
Differential expression of steroidogenic genes in *P. olivaceus* and *C. semilaevis*, as (**a**) wildtype female ovary (FO) vs. wildtype male testis (MT) and gynogenetic female ovary (GFO) vs. gynogenetic neo-male testis (GNMT) of *P. olivaceus*; (**b**) brain (B) and gonad (O/T) of gynogenetic *P. olivaceus*; (**c**) four HPG/L axis tissues of male and female *C. semilaevis*, as L-liver, B-brain, P-pituitary, O/T-ovary/testis; (**d**) brain (B) and gonad (O/T) of female, male and neo-male *C. semilaevis*. The Heatmaps were represented with the log_2_(FC) values of each gene. * indicates *p* < 0.05, ** indicate *p* < 0.01, *** indicate *p* < 0.001.

The steroidogenesis during sexual development in teleosts is essentially regulated by differential expression of several steroidogenic enzymes. Through comparative transcriptome analysis of brains and gonads, most steroidogenic genes represented conserved expression patterns between *P. olivaceus* and *C. semilaevis* (Table S4). In gonad tissues, the most differentially expressed genes (DEGs) were obtained between the ovaries and testis (male/neo-male), while only a few DEGs were found between the normal male and sex reversed neo-male testes (Figure 5 and Figure S3). For example, in the two gynogenetic *P. olivaceus* gonad transcriptomes, *cyp11a*, *cyp11c*, *cyp17a1* and *hsd3b1* had testis biased expression (Figure 5a,b), while *cyp19a1a*, *cyp26a1*, *hsd17b1* and *hsd17b12a* had ovary biased expression (Figure 5a,b). In addition, the expression of steroidogenic genes between the gonads of the wide type and gynogenetic *P. olivaceus* was also similar (Figure 5a), with only *cyp26a1* being highly expressed in the gynogenetic female ovaries but not in the wild type *P. olivaceus* gonads (Figure 5a), indicating consistent steroid biosynthesis functions between wild type and gynogenetic individuals. Similarly, in the *C. semilaevis* gonads, there were also conserved expression patterns of steroidogenic genes. For example, *star-3*, *cyp11a*/*c*, *cyp17a1*/*a2*, *cyp21a*, *cyp26a1*, *hsd3b1*/*b7*, *hsd11b2*, *hsd20b2*, *esr1* and *esr2a* all had testis biased expression in the two transcriptomes, while only *hsd17b12a* had ovary biased expression (Figure 5c,d). Interestingly, there were more testis biased than ovary biased steroidogenic genes in both the *P. olivaceus* and *C. semilaevis* gonads, which was in line with our previous finding [34], suggesting significant male sex steroid hormone activities in both the *P. olivaceus* and *C. semilaevis* testis. Furthermore, there was less than expected DEGs between the normal male and sex reversed neo-male testis of both *P. olivaceus* and *C. semilaevis*. For example, only *cyp19a1b* was differentially expressed between the neo-male and male testis of *P. olivaceus*, and no DEGs were present between the neo-male and male testis of *C. semilaevis* (Figure 5a,d). This was probably due to the similar steroid hormone biosynthesis process between the mature male and neo-male testis, which could both develop functional sperms. Therefore, other functional pathways may warrant further investigation to ascertain specific differences in testis development and spermatogenesis between male and neo-male *P. olivaceus* and *C. semilaevis*. In addition, only a few steroidogenic genes were differentially expressed between the male and female brains of *P. olivaceus* and *C. semilaevis*. For example, four genes were differentially expressed between the brains of male and female gynogenetic *P. olivaceus*, with *cyp21a* and *hsd3b1* being male brain biased, and *cyp19a1a* and *hsd17b1* being female brain biased (Figure 5b); only *star-3* was differentially expressed between the brains of male and female *C. semilaevis* (Figure 5c).

To further understand the function of steroidogenic genes, GO and KEGG enrichment of their homologous in model organisms (human and zebrafish) were analyzed with various biological processes (BPs), molecular functions (MFs) and cellular components (CCs) (Figure S4). Specifically, the steroidogenic genes were enriched in five BPs, including steroid biosynthetic process, estrogen biosynthetic process, response to estrogen, glucocorticoid biosynthetic process and sterol metabolic process (Figure S4a); five MFs contained estradiol 17-beta-dehydrogenase activity, testosterone dehydrogenase (NAD+) activity, steroid binding, aromatase activity and steroid hydroxylase activity (Figure S4b), as well as one CC of the endoplasmic reticulum membrane (Figure S4c). Moreover, the steroidogenic genes were enriched in five KEGG pathways, including steroid hormone biosynthesis, metabolic pathways, retinol metabolism, ovarian steroidogenesis, aldosterone synthesis and secretion (Figure S4d). Therefore, these genes were supported to participate in various processes of steroid hormone biosynthesis. For example, *cyp19a1a* and several *hsd17b* (*1*, *2*, *8*, *11*, *12*) genes were mainly associated with the estrogen biosynthetic process and estradiol 17-beta-dehydrogenase activity (Figure S4a,b), which was in agreement with their ovary biased expression pattern (Figure 5). Moreover, *cyp11a*/*c*, *cyp21a*, *hsd3b1*/*b2* and *hsd11b1*/*b2* genes participated in both glucocorticoid biosynthetic processes and aldosterone synthesis and secretion (Figure S4a,d), indicating their possible crosstalk in different steroid hormone biosynthesis.

### 2.5. Coordinated Interaction of Steroidogenic Pathway in P. olivaceus and C. semilaevis

**Figure 6:**
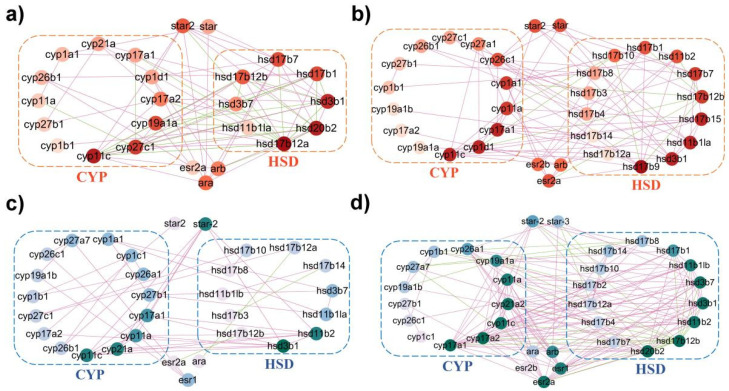
Network analysis of steroidogenic enzymes in *P. olivaceus* and *C. semilaevis* with Pearson’s correlation coefficient (PCC) of FPKM values. (**a**,**b**) represent networks in *P. olivaceus* corresponding to Figure 5a,b; (**c**,**d**) represent networks in *C. semilaevis* corresponding to Figure 5c,d. Red lines indicate positive correlation of PCC > 0.8 and green lines indicate negative correlation of PCC < −0.8.

To investigate the possible cascade regulation of steroidogenic genes, the STRING database was employed to show that the steroid biosynthesis proteins could interact directly with each other in model organisms (Figure S5). For example, *cyp1a*, *cyp11b*/*c*, *cyp17a1*, *cyp19a1*, *hsd3b1* and several *hsd17b* genes were highly interactive with other steroidogenic genes in zebrafish and humans (Figure S5). In addition, with PCC > 0.8 as highly correlation, interaction networks revealed a high level of coordinated regulation among steroidogenic genes of *P. olivaceus* and *C. semilaevis*, with more positive correlation than negative correlation (Table S5). For instance, in *P. olivaceus*, *cyp11c*, *cyp17a1*, *cyp19a1a*, *hsd3b1*, *hsd20b* and *hsd17b* genes appeared to interact greatly with other steroidogenic genes (Figure 6a,b), while *cyp11a*/*c*, *cyp17a1*/*a2*, *cyp21a*, *hsd3b1*, *hsd11b2* and *hsd20b2* were the most interactive genes in *C. semilaevis* (Figure 6c,d). Most of these genes represented testis biased expression (Figure 5), indicating their possible essential coordinated roles in testicular development and spermatogenesis. Moreover, in *P. olivaceus* and *C. semilaevis*, the sex hormone receptor genes, *esr* and *ar*, also represented high connectivity with other genes (Figure 6), which was in accordance with their transcription factor function. For example, in the steroidogenic pathway, both *esr* and *ar* may have direct interactions with *cyp19a1* genes, and therefore, participate in the regulation of the transcriptional activity of *cyp19a1* [31].

**Figure 7:**
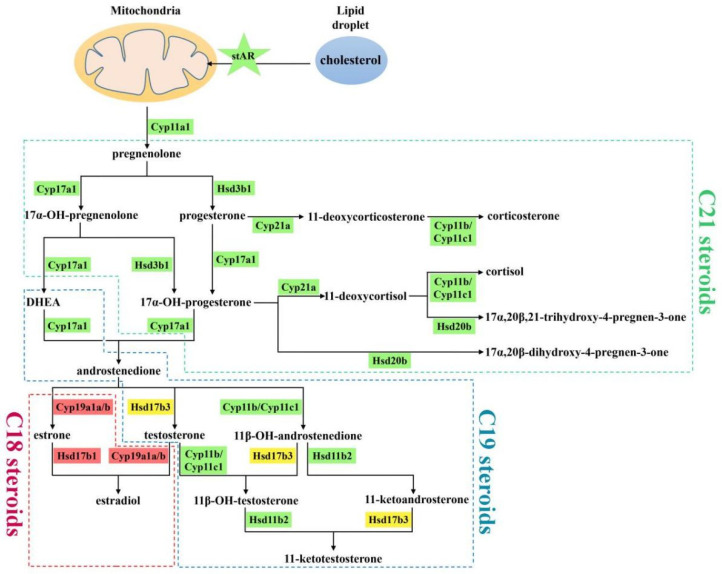
Putative steroid hormone biosynthesis pathway in *P. olivaceus* and *C. semilaevis*. Genes with green background represent higher expression in testis, genes with red background represent higher expression in ovaries, and controversial expression genes were represented with yellow background.

In the sophisticated reaction cascade of steroid hormone biosynthesis in the gonads, *star* assists the transport of precursor cholesterol from the outer to the inner mitochondrial membrane (Figure 7) [4]. In *P. olivaceus* and *C. semilaevis*, *star* (*star-2*/*3*) and *star2* were highly expressed in the testis of both species (Figure 5 and Figure 7), indicating their functional conservation in the production of male-specific steroid hormones. In Nile tilapia (*Oreochromis niloticus*), androgen generation during spermatogenesis may require the involvement of *star*, which was proven to be primarily expressed in the testis [32]. Interestingly, even with *star-1* not expressed in the gonads of *C. semilaevis*, *star-2* and *star-3* were highly expressed in the testis of *C. semilaevis* (Figure 5c,d), suggesting their diverged function in *C. semilaevis* testis development and spermatogenesis. In mitochondria, cyp11a is responsible for subsequently catalyzing the side-chain hydroxylation and cleavage of cholesterol to pregnenolone (Figure 7) [35], which together with star for cholesterol mobilization, controls the rate of steroid hormone biosynthesis [22]. *Cyp11a* was greatly expressed in both the male and neo-male testes of *P. olivaceus* and *C. semilaevis* (Figure 5 and Figure 7), and it interacted with *star* in *C. semilaevis* network (Figure 6c), indicating that the steroidogenesis is officially initiated under the action of *star* and *cyp11a*, which may be more closely related to testicular development in teleost species [36].

Cyp17a and hsd3b are important enzymes as the second step of steroidogenesis involving the conversion of pregnenolone into 17α-OH-pregnenolone and progesterone, and later to androstenedione [12,14,37] (Figure 7). *Cyp17a1* and *cyp17a2* were reported to be highly expressed in the *P. olivaceus* testis and ovary [30], and *cyp17a1* and *hsd3b1*/*b7* were all strongly expressed in the testis of both *P. olivaceus* and *C. semilaevis* (Figure 5). Here, in the networks, *cyp17a1*/*a2* and *hsd3b1* were closely associated with each other (Figure 6), which corresponded to their catalytic function of transforming pregnenolone into androstenedione (Figure 7). Moreover, *cyp17a1*/*a2* and *hsd3b1*/*b7* also strongly interacted with testis biased *cyp11a*/*c*, *cyp21a*, *hsd20b*, as well as negatively correlated with ovary biased *cyp19a1a* and *hsd17b* genes (Figure 6), indicating their essential regulatory interaction in the steroidogenesis cascade. For example, *hsd20b* has been demonstrated to be associated with 17α, and 20β-DP synthesis [38] as well as testicular development [39]. Here *hsd20b* was dominant in testicular expression in both *P. olivaceus* and *C. semilaevis* (Figure 5) and has a similar interaction pattern as *hsd3b1* with other steroidogenic genes (Figure 6), corresponding to the sequential interaction of *hsd3b1* and *hsd20b* along with the biosynthesis of the C21 steroid (Figure 7).

Subsequently, cyp11c and hsd11b participate in the production of 11-ketotestosterone (11-KT) precursors as well as the biosynthesis of cortisol in teleosts (Figure 7); therefore, they could function essentially in the crosstalk between the HPG and HPI axes [40,41]. In addition, steroid 21-hydroxylase (cyp21a) has a crucial role in the formation of 11-deoxycorticosterone and 11-deoxycortisol [6]. Together with *cyp11c*, *hsd11b* was proven to synthesize 11-KT from testosterone (T) and interact with *cyp21a* to promote the formation of cortisol and corticosterone (Figure 7) [42], which was consistent with the reaction network results in *P. olivaceus* and *C. semilaevis* that *cyp11c* was strongly associated with *hsd11b* and *cyp21a*, respectively (Figure 6). All these three genes were strongly expressed in the male and neo-male testis of *P. olivaceus* and *C. semilaevis* (Figure 5), indicating their essential roles in androgen related hormones biosynthesis. Interestingly, *hsd11b1la* was highly expressed in the *P. olivaceus* testis but not in the *C. semilaevis* testis, while *hsd11b2* was greatly expressed in the *C. semilaevis* testis but not in the *P. olivaceus* testis (Figure 5), suggesting their putatively lineage-specific function in different teleost species.

On the other side, aromatase (cyp19a1a) catalyzes the production of aromatic C18 estrogens from C19 androgens, for instance, from androstenedione into estrone (E1) and from T into estradiol (E2) (Figure 7) [28,43]. The expression of *cyp19a1a* was higher in the ovary than in the testis, while *cyp19a1b* was notably highly expressed in the brain than in the gonads as well as highly expressed in neo-male brains of *P. olivaceus* and *C. semilaevis* (Table S4). A similar *cyp19a1* expression pattern was also reported in other teleost species, such as in Clupeocephala and zebrafish [44,45], which suggested that *cyp19a1b* is relevant to brain development and repair in teleost lineages [46]. Together with *cyp19a1a*, *hsd17b1* is responsible for the generation of E2 [47], and the network results were consistent with this collaboration, showing a strong correlation between *cyp19a1a* and *hsd17b* genes (Figure 6a,c,d). *Hsd17b1* was proven to be highly expressed in the ovary of *P. olivaceus* [24], but over-expressed in the testis of *C. semilaevis* (Figure 5). *Hsd17b3* is involved in T production [20], with the expression of *hsd17b3* higher in the testis of *O. niloticus* and *P. olivaceus* [24,48]. In this study, *hsd17b3* was not differentially expressed in either *P. olivaceus* or *C. semilaevis* gonads (Figure 5), which requires further experimental verification. Moreover, *hsd17b12a* and *hsd17b12b* can accelerate the conversion of E1 into E2 and promote estrogen synthesis [49]. Only *hsd17b12a* but not *hsd17b12b* was highly expressed in the ovary of *P. olivaceus* and *C. semilaevis* (Figure 5), suggesting their functional divergence. This was in line with the previous report that *hsd17b12* was distinctly expressed in the *P. olivaceus* ovary [24]. All in all, these interactive networks indicate the cascading effect of key steroidogenic enzymes and warrant further investigation with a global biological impact in teleost lineages.

## 3. Materials and Methods

### 3.1. Characterization of Steroidogenic Genes in P. olivaceus and C. semilaevis

The coding sequences (cds) and amino acid (aa) sequences of steroidogenic genes from selected vertebrates, including tetrapods human (*Homo sapiens*), mouse (*Mus musculus*), chicken (*Gallus gallus*), frog (*Xenopus tropicalis*) and fishes spotted gar (*Lepisosteus oculatus*), zebrafish (*Danio rerio*), stickleback (*Gasterosteus aculeatus*), medaka (*Oryzias latipes*), fugu (*Takifugu rubripes*), were retrieved from NCBI (http://www.ncbi.nlm.nih.gov (accessed on 15 May 2021)) and Ensembl (http://asia.ensembl.org (accessed on 15 May 2021)) database. These sequences were used as queries to search for their homologous genes in the genomes of *P. olivaceus* and *C. semilaevis* by BLASTN (e-value = 1 × 10^−5^), respectively.

Multiple Em for Motif Elicitation (MEME, https://meme-suite.org/meme (accessed on 7 July 2021)) program was applied to evaluate the conserved motifs with the multiple sequence alignment of the aa sequences, with the parameters of any number of repetitions, optimum width of motifs from 6 to 200 with 6 motifs for *cyp* genes, 12 motifs for *hsd* genes, 12 motifs for *star*, *ar* and *esr*, respectively. The Gene Structure Display Sever (GSDS 2.0, http://gsds.gao-lab.org (accessed on 7 July 2021)) was used to visualize the exon-intron structure of steroidogenic genes. The gene structure and conserved motifs were visualized using TBtools V1.09 [50].

### 3.2. Phylogenetic Analysis of Steroidogenic Genes

The multiple sequence alignment was performed using MUSCLE [51] with the aa sequences of *cyp*, *hsd*, *star*, *ar* and *esr* genes, respectively. The Maximum Likelihood (ML) phylogenetic trees were generated using the IQ-Tree (http://iqtree.cibiv.univie.ac.at/ (accessed on 4 June 2021 )) with a bootstrap of 1000 replicates and were visualized by the iTOL (https://itol.embl.de/ (accessed on 6 June 2021)). The best models selected for phylogeny construction were the JTT + F + I + G4 model for *cyp* genes, the VT + F + G4 model for *hsd* genes, the JTT + G4 model for *star*, the JTT + F + I + G4 model for *ar*, and the JTT + F + I + G4 model for *esr*.

### 3.3. Synteny Analysis of Steroidogenic Genes

Seven pairs of steroidogenic genes, which were putatively originated from the teleost specific genome duplication (TSGD) event, were selected for synteny analysis, including *star*/*star2*, *cyp11a*/*cyp11b*(*c*), *cyp17a1*/*cyp17a2*, *cyp19a1a*/*cyp19a1b*, *hsd17b12a*/*hsd17b12b*, *esr2a*/*esr2b* and *ara*/*arb*. With the Ensembl and Genomicus (https://www.genomicus.bio.ens.psl.eu/genomicus-105.01/cgi-bin/search.pl (accessed on 25 July 2021)) database, the genomic locations of upstream and downstream genes from 10 selected vertebrates, including human (*H. sapiens*), mouse (*M. musculus*), chicken (*G. gallus*), frog (*X. tropicalis*), spotted gar (*L. oculatus*), Chinese tongue sole (*C. semilaevis*), zebrafish (*D. rerio*), medaka (*O. latipes*), stickleback (*G. aculeatus*) and fugu (*T. rubripes*), were collected and sorted. The genomic structure of *P. olivaceus* genes was obtained from the NCBI database.

### 3.4. Molecular Evolution Analysis

Molecular evolution analysis was performed with PAML [52] in EasyCodeML V1.21 [53] to compare the evolution rates of steroidogenic genes in teleost lineages, including 10 teleost species as Japanese flounder (*P. olivaceus*), Chinese tongue sole (*C. semilaevis*), zebrafish (*D. rerio*), stickleback (*G. aculeatus*), medaka (*O. latipes*), fugu (*T. rubripes*), Asian sea bass (*Lates calcarifer*), platyfish (*Xiphophorus maculatus*), tilapia (*Oreochromis niloticus*) and turbot (*Scophthalmus maximus*). Firstly, the ratio of non-synonymous to synonymous substitutions (dN/dS, ω) and the likelihood ratio tests (LRTs) was employed to evaluate the selective pressure in each gene. Six site model (SM) tests were used to evaluate the positive selection in each codon: M0 assumes to have the same ω for all codons, M3 assumes the ω of all codons showing a simple discrete division trend; M1a assumes only conservative sites (0 < ω < 1) and neutral sites (ω = 1) for all codons, while M2a is considered to increase the existence of positive sites (ω > 1) for all codons on the basis of M1a; the ω of all codons in M7 are assumed to belong to the matrix (0, 1) with a beta distribution, while M8 adds another type of ω (ω > 1) on the basis of M7. LRTs were used to judge whether the paired models (M0 vs. M3; M1a vs. M2a; M7 vs. M8) are significantly different [52], and to estimate whether there are positively selected sites (PSSs) (M2a vs. M1a and M8 vs. M7) under the premise of a significant *p*-value (*p* < 0.05). For branch model (BM) tests, firstly, the *P. olivaceus* branch was labeled as the foreground branch (ω1) and the other teleosts as the background branch (ω0) to compare the evolution rates of steroidogenic genes in *P. olivaceus* against other teleosts; secondly, for the putative duplication originated genes, each gene cluster was labeled as the foreground branch (ω1) to compare it with the other branch (ω0) in teleost lineages.

### 3.5. Expression of Steroidogenic Genes through Transcriptomic Analysis

To investigate the expression profiles of steroidogenic genes, four RNA-seq datasets from *P. olivaceus* and *C. semilaevis* were obtained from NCBI. For *P. olivaceus*, one dataset was from the gonad tissues of 1.5-year-old wildtype female ovary (FO), wildtype male testis (MT), gynogenetic female ovary (GFO), gynogenetic neo-male testis (GNMT) (PRJNA639001) [54], and the other dataset was from the brain and gonad tissues of 1.5-year-old gynogenetic *P. olivaceus* (PRJNA764760) [34]. Two transcriptomes for *C. semilaevis* were also employed, such as liver, brain, pituitary and gonad tissues of 2-year-old male and female individuals (PRJNA413516) [55], as well as the brain and gonad tissues of 1-year-old male, female and pseudo-male individuals (PRJNA480118) [27].

For transcriptome analysis, the fastq files were first optimized by trimmomatic [56], to remove adapters, low-quality reads, and the reads with less base numbers. Next, the read mapping and quantification were performed with the Hisat and StringTie pipeline [57], and the Fragments Per Kilobase of exon model per Million mapped fragments (FPKM) values were extracted for each steroidogenic gene. Differentially expressed genes (DEGs) between groups were determined by edgeR [58]. Any pair-wise genes with log_2_|fold change| (log_2_FC) > 2, adjusted *p*-value < 0.05 and at least one gene with FPKM >1 was identified as the DEG. TBtools were utilized to draw heat maps with both log_2_(FPKM + 1) and log_2_FC values. GO and KEGG annotation of steroidogenic genes were obtained from DAVID (https://david.ncifcrf.gov/ (accessed on 8 September 2021)) for humans and zebrafish. Redundancy (semantically similar terms) and pathways involving a low number of genes (<3) were removed.

### 3.6. Protein-Protein Interaction and Co-Expression Analysis

Protein-protein interaction (PPI) networks of steroidogenic genes in humans and zebrafish were analyzed using the online site STRING 11.5 (https://string-db.org/ (accessed on 14 August 2021)) with the parameter, minimum required interaction score, set to 0.7. For *P. olivaceus* and *C. semilaevis*, OmicShare (https://www.omicshare.com/ (accessed on 17 August 2021)) was employed for the Pearson’s correlation coefficient (PCC) analysis of FPKM values between steroidogenic genes with a PCC threshold of 0.8 from the transcriptome analysis. Cytoscape [59] was employed to illustrate the networks according to the PCC values.

## 4. Conclusions

In summary, steroidogenic genes were comprehensively characterized in *P. olivaceus* and *C. semilaevis*, with *cyp19a1a*/*cyp19a1b*, *hsd17b12a*/*hsd17b12b*, *ara*/*arb* and *esr2a*/*esr2b* originating from the TSGD, and *star*/*star2* and *cyp17a1*/*cyp17a2* putatively evolving independently. The expression profile and network analysis indicated the key factors functioning essentially during the steroidogenesis of *P. olivaceus* and *C. semilaevis*. Specifically, *star*, *cyp11a*/*c*, *cyp17a1*/*a2*, *cyp21a*, *hsd3b1*/*b7*, *hsd11b* and *hsd20b* were primarily expressed in the testis of *P. olivaceus* and *C. semilaevis*, whereas *cyp19a1a* and *hsd17b* genes were highly expressed in the ovaries, which constitute the coordinated regulatory network during the steroidogenic process. Interestingly, only a few steroidogenic genes were differentially expressed between male and neo-male testis, which warrants more functional verification. Investigation of other functional pathways, such as the piRNA pathway [60], may help to explain the specific difference in testis development and spermatogenesis between male and neo-male *P. olivaceus* and *C. semilaevis* in the future. All these findings will provide significant insights into the steroidogenic enzyme cascade of the teleost lineages and may contribute to reproductive manipulation in aquaculture.

## Data Availability

The transcriptome datasets used in this study can be found in the NCBI Sequence Read Archive (SRA) BioProject as PRJNA639001 and PRJNA764760 for *P. olivaceus*, and PRJNA413516 and PRJNA480118 for *C. semilaevis*.

## Supplementary Materials

The following supporting information can be downloaded at: https://www.mdpi.com/article/10.3390/ijms23105520/s1.
