# Supplementary material for: The Comparative Survey of Coordinated Regulation of Steroidogenic Pathway in Japanese Flounder (Paralichthys olivaceus) and Chinese Tongue Sole (Cynoglossus semilaevis)
Source: Int J Mol Sci. 2022 May 15;23(10):5520. doi: 10.3390/ijms23105520 (PMC9141715; doi:10.3390/ijms23105520)
Supplement: Supplementary file 1 [file ijms-23-05520-s001.zip › Table S1.Copy numbers.pdf]

Gene		*Hsa*	*Mmu*	*Gga*	*Xtr*	*Loc*	*Dre*	*Gac*	*Ola*	*Tru*	*Cse*	*Pol*
Star	*star*	1	1	1	1	1	1	1	1	1	3	1
	star2	0	0	0	0	1	1	1	1	1	1	1
Cyp	*cyp1a*	2	2	2	1	1	1	1	1	2	1	1
	cyp1b	1	1	1	1	1	1	1	1	1	1	1
	cyp1c	0	0	1	1	1	2	2	1	2	2	0
	cyp1d	0	0	0	1	1	1	1	1	0	0	1
	cyp11a	1	1	1	1	1	2	1	1	1	1	1
	cyp11c	2	2	0	1	0	1	1	1	1	1	1
	cyp17a	1	1	1	1	2	2	2	2	2	2	2
	cyp19a	1	1	1	1	1	2	2	2	1	2	2
	cyp21a	1	1	1	1	0	1	1	1	1	1	1
	cyp26a	1	1	1	1	1	1	1	1	1	1	1
	cyp26b	1	1	1	1	1	1	1	1	1	1	1
	cyp26c	1	1	1	1	1	1	1	1	1	1	1
	cyp27a	1	1	1	1	1	4	1	1	1	1	1
	cyp27b	1	1	0	1	1	1	1	1	1	1	1
	cyp27c	1	0	1	1	0	1	1	1	1	1	1
Hsd	*hsd3b*	3	7	3	2	2	3	2	3	2	2	2
	hsd11b1	2	1	3	3	1	2	2	2	1	2	1
	hsd11b2	1	1	1	1	1	1	1	1	1	1	1
	hsd17b	14	15	11	12	6	11	9	10	9	10	11
	hsd20b2	0	0	0	0	1	1	1	1	1	1	1
Esr	*esr1*	1	1	1	1	1	1	1	1	1	1	1
	esr2	1	1	1	1	1	2	2	2	2	2	2
Ar	*ar*	1	1	1	1	1	1	2	2	2	2	2
**Total**		**39**	**42**	**35**	**37**	**29**	**46**	**40**	**41**	**38**	**42**	**39**
