# Supplementary figures and images for: The Comparative Survey of Coordinated Regulation of Steroidogenic Pathway in Japanese Flounder (Paralichthys olivaceus) and Chinese Tongue Sole (Cynoglossus semilaevis)
Source: Int J Mol Sci. 2022 May 15;23(10):5520. doi: 10.3390/ijms23105520 (PMC9141715; doi:10.3390/ijms23105520)

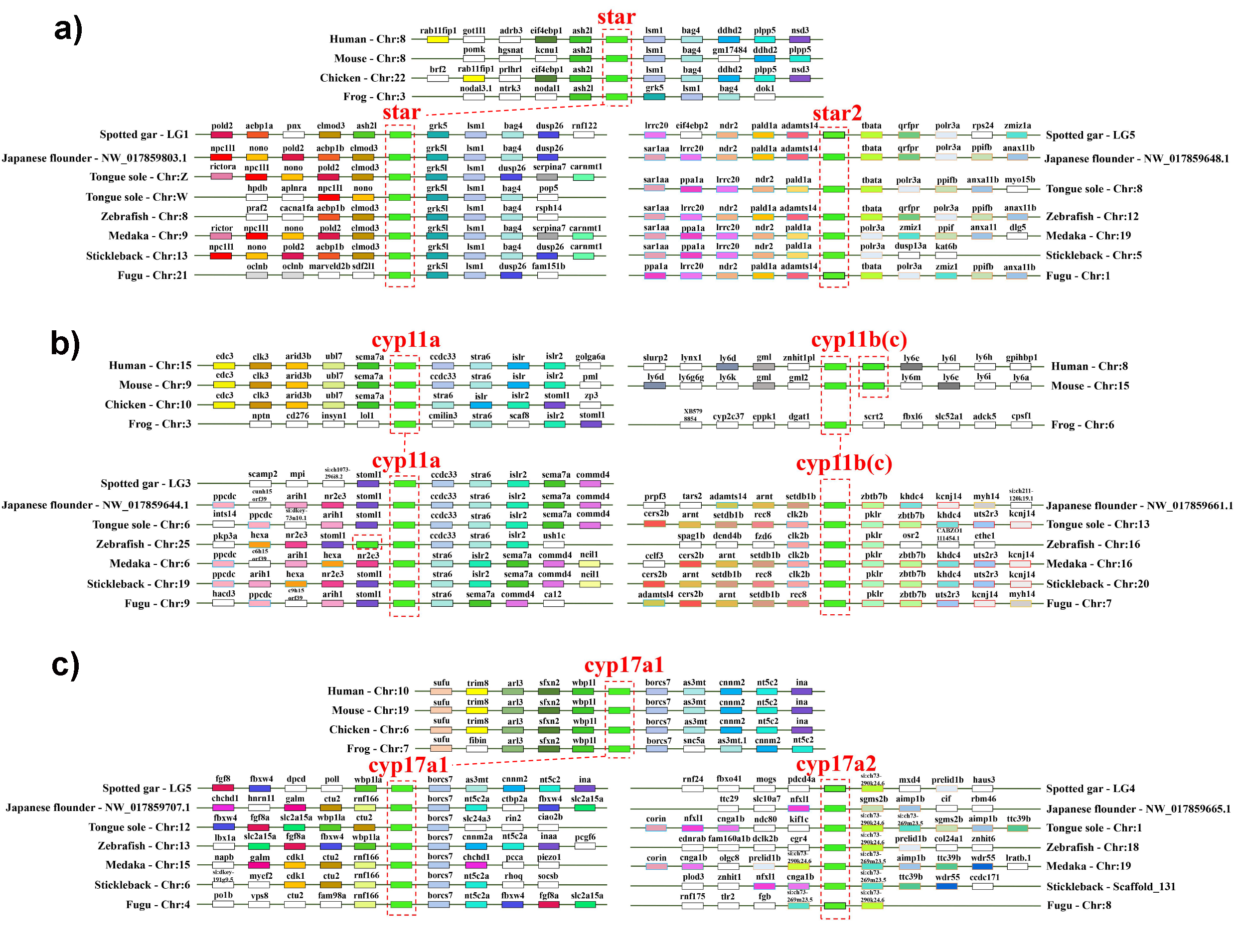

Supplement: Supplementary file 1 [file ijms-23-05520-s001.zip › Fig.S1.Synteny.tiff]

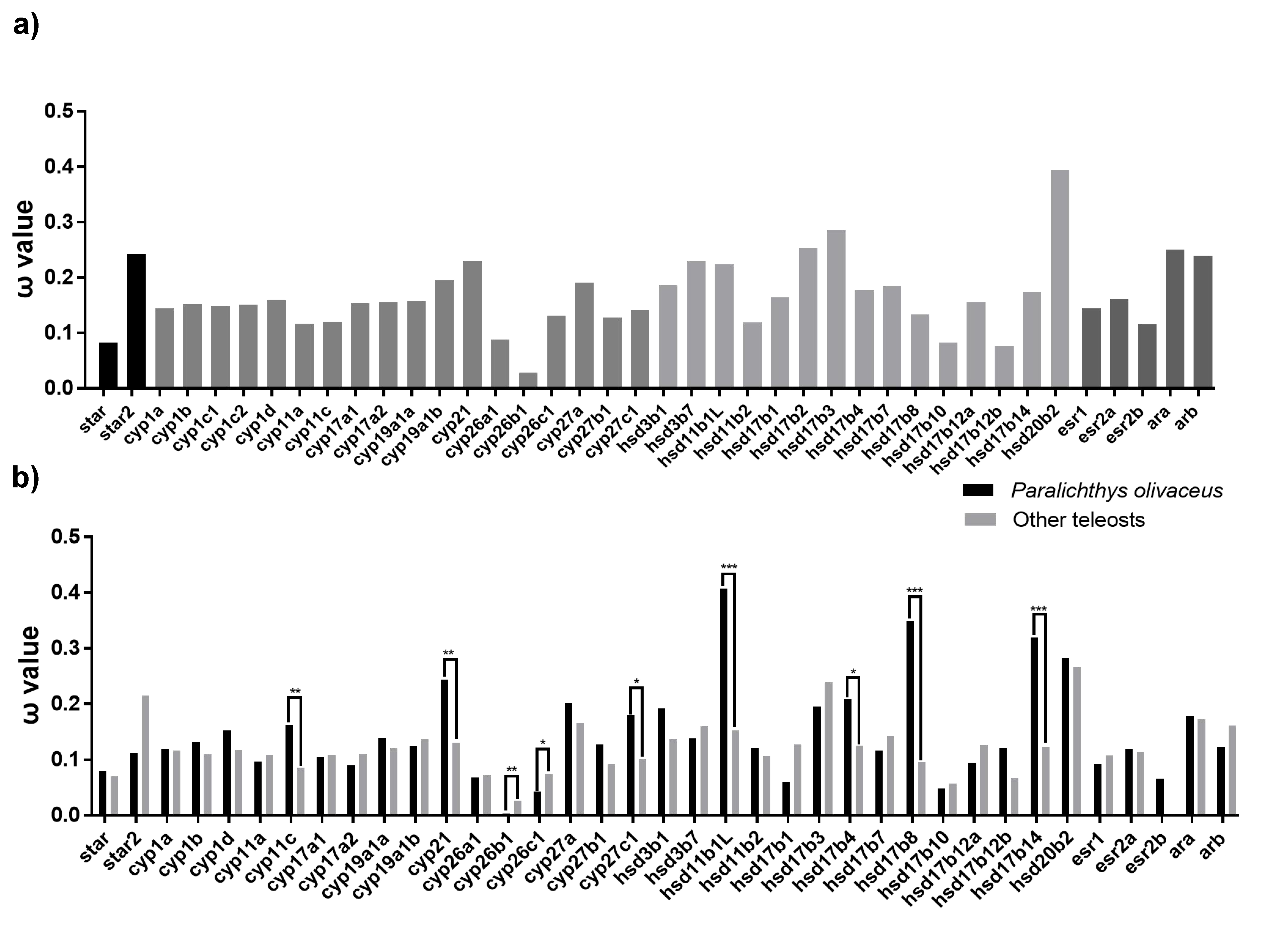

Supplement: Supplementary file 1 [file ijms-23-05520-s001.zip › Fig.S2.PAML.tiff]

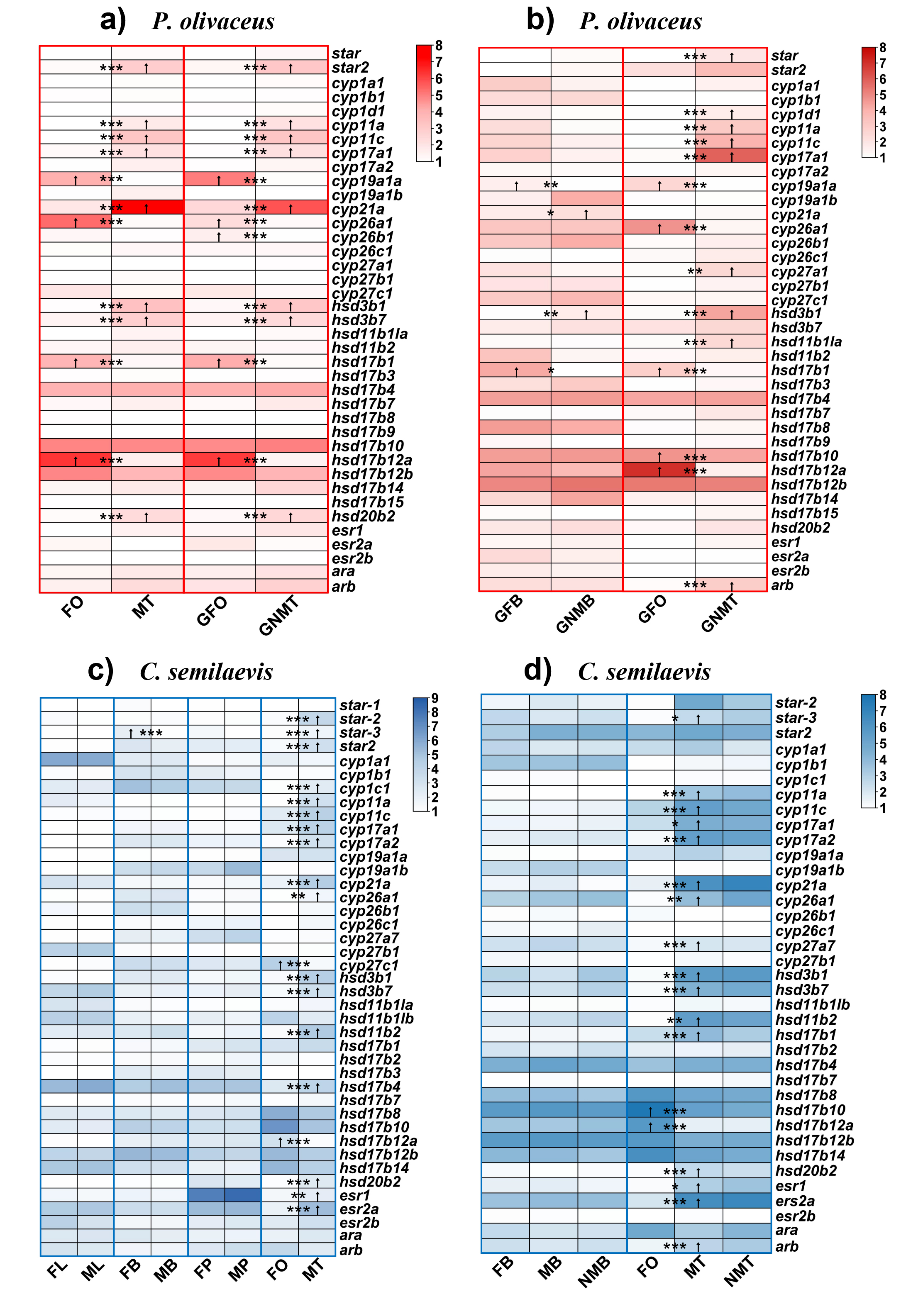

Supplement: Supplementary file 1 [file ijms-23-05520-s001.zip › Fig.S3.HeatMap-FPKM.tiff]

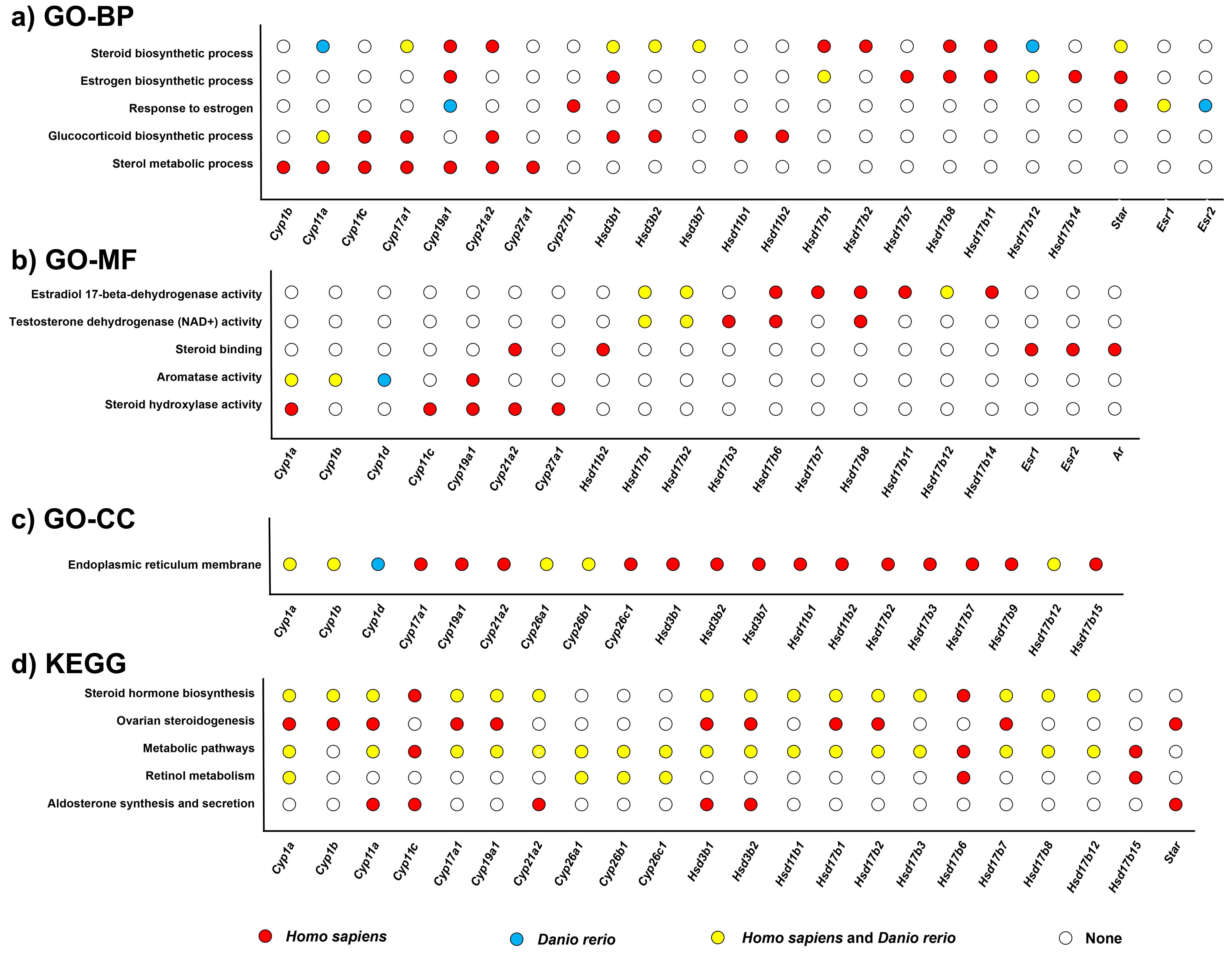

Supplement: Supplementary file 1 [file ijms-23-05520-s001.zip › Fig.S4.GO-KEGG2.tif]

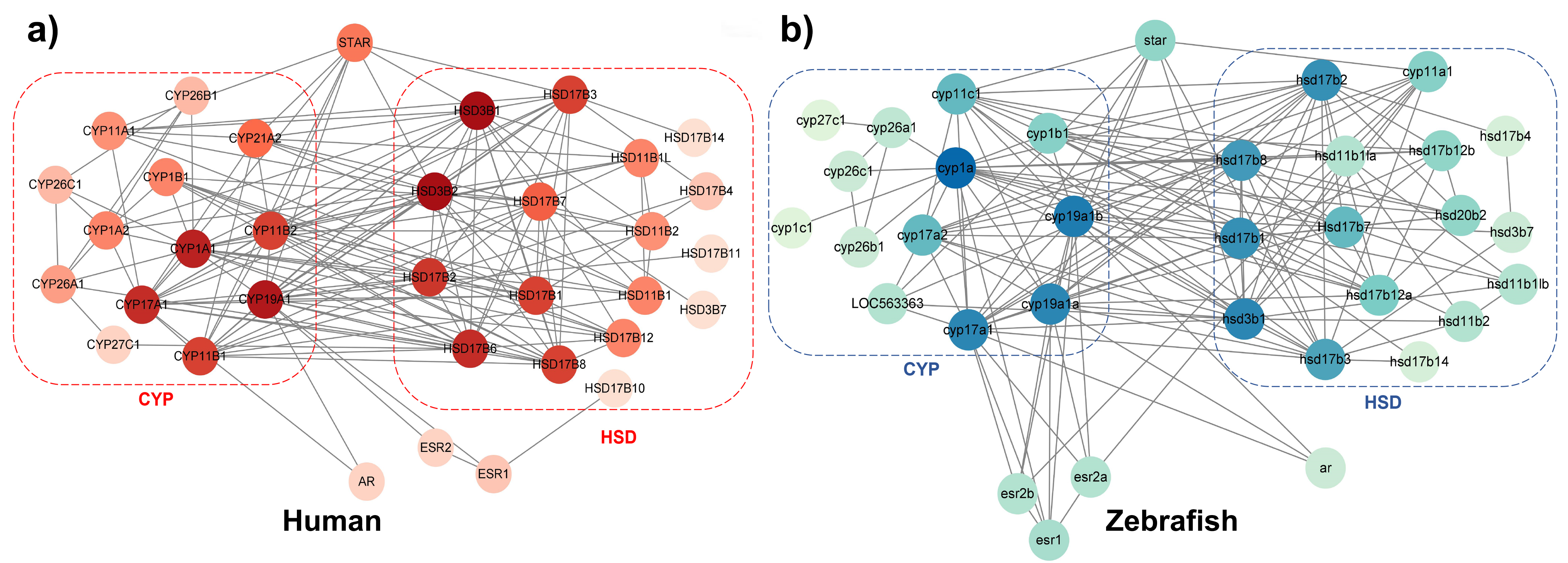

Supplement: Supplementary file 1 [file ijms-23-05520-s001.zip › Fig.S5.PPI-Hsa&Dre2.tif]
